# Involvement of the opportunistic pathogen *Aspergillus tubingensis* in osteomyelitis of the maxillary bone: a case report

**DOI:** 10.1186/1471-2334-13-59

**Published:** 2013-02-01

**Authors:** Erik Bathoorn, Natalia Escobar Salazar, Shahrzad Sepehrkhouy, Martin Meijer, Hans de Cock, Pieter-Jan Haas

**Affiliations:** 1 Department of Medical Microbiology, University Medical Center Utrecht, Postbox 85500, Utrecht, The Netherlands; 2 Department of Pathology, University Medical Center Utrecht, Utrecht, The Netherlands; 3 Institute of Biomembranes, Faculty of Science, Utrecht University, Utrecht, The Netherlands; 4 Applied and Industrial Mycology/Identification Service, CBS-KNAW Fungal Biodiversity Centre, Utrecht, the Netherlands

**Keywords:** *Aspergillus tubingensis*, *Aspergillus* section *nigri*, Invasive mould infection, Species identification

## Abstract

**Background:**

*Aspergillus tubingensis* is a black *Aspergillus* belonging to the *Aspergillus* section *Nigri,* which includes species that morphologically resemble *Aspergillus niger*. Recent developments in species determination have resulted in clinical isolates presumed to be *Aspergillus niger* being reclassified as *Aspergillus tubingensis* by sequencing*.* We present a report of a patient with an osteomyelitis of the maxillary bone with a probable invasive *Aspergillus tubingensis* infection.

**Case presentation:**

We describe an immune compromised patient suffering from osteomyelitis of the maxillary bone after tooth extraction. The osteomyelitis probably resulted in dentogenic pansinusitis presenting as an acute ethmoiditis. Histologic examination of biopsy samples showed osteomyelitis, and inflammation of the surrounding connective tissue. Cultures of the alveolar wound grew *Aspergillus tubingensis.* The patient was treated with liposomal amphoterocin B, which was changed to oral treatment with voriconazole based on susceptibility testing (MIC for voriconazole was 1 μg/ml).

**Conclusion:**

This case shows that *Aspergillus tubingensis* may have the potential to cause severe invasive infections in immunocompromised hosts. A larger proportion of *Aspergillus tubingensis* isolates are less susceptible to azoles compared to *Aspergillus niger*. Therefore, correct species identification and susceptibility testing is crucial for the choice of anti-fungal treatment, screening of azole resistance, and characterization of the pathogenic potential of the various species within *Aspergillus* section *Nigri.*

## Background

*Aspergillus tubingensis* is a black *Aspergillus* species described by Raoul Mosseray in 1934 [1]. This species is found all over the world, grows predominantly on dead plant material, and is food associated [2]. *A. tubingensis* belongs to the *Aspergillus* section *Nigri*, which also includes *A. niger*, and *A. awamori* amongst a total of 25 species [2-4]. The species belonging to the *Aspergillus* section *Nigri* are phylogenetically closely related. This makes the taxonomy and species determination difficult. In general, species can be distinguished via a polyphasic approach using morphology, biochemical properties, and molecular data [3,5]; however, most of the species belonging to the *Aspergillus* section *Nigri* are morphologically indistinguishable. Several species do have some distinct biochemical properties: nutritional growth conditions and hydrolase differences between the species have been described [6]. Furthermore, production of secondary metabolites is often unique for a species within *Aspergillus* section *Nigri*, and could be used for identification [2], but it is not yet possible to differentiate the species solely on metabolic properties.

Due to the above mentioned difficulties in discrimination of species, the most commonly used method for species identification of *Aspergillus* section *Nigri* is sequencing. Using calmodulin or β-tubulin data sequencing data, all *Aspergillus* section *Nigri* species can be clearly distinguished [3]. The development of these molecular diagnostic tools has facilitated correct species determination of black *Aspergilli*.

In this report, we describe a patient with a probable invasive infection with *A. tubingensis,* and discuss the clinical importance of *A. tubingensis* and its correct species determination.

## Case presentation

A 19-year-old male patient born in the Netherlands (Moroccan parents) presented with fever, rhinitis and progressive pain behind the left eye. His recent medical record included a second non-myeloablative stem cell transplantation for graft failure resulting in relapse of paroxysmal nocturnal haemoglobinuria 3 weeks prior to presentation. He had undergone a maxillary tooth extraction 47 days prior to presentation. The alveolar wound did not heal due to osteomyelitis, for which he had a biopsy of the maxillary bone 23 days prior to presentation. Histological examination of the biopsy samples showed necrotic bone tissue. The surrounding connective tissue was infiltrated by leukocytes. Yeast cells, and focal bacterial colonies were noted. He was treated empirically with amoxicillin/clavulanic acid orally, which had been started 3 days prior to presentation, and the immunosuppressive drugs cyclosporine, mycophenolic acid, and prednisone. With this treatment, the patient was clinically deteriorating.

**Figure 1:**
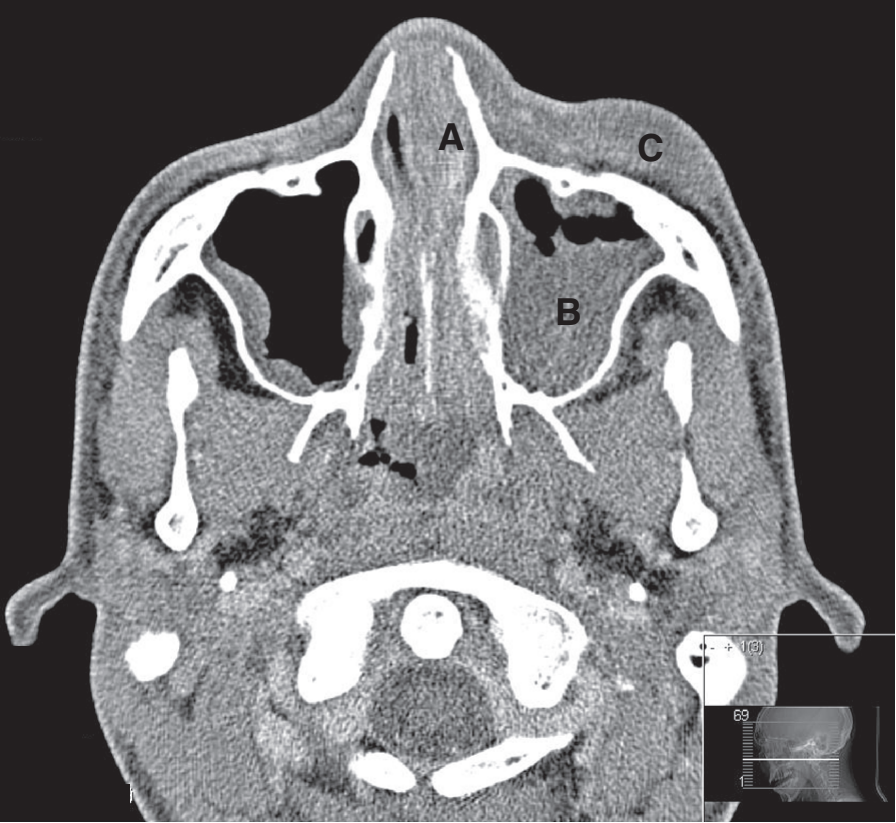
Computed tomography of the head showing A: total opacification of the ethmoid sinus; B: swollen mucosa of the maxillary sinus; C: pre-orbital swelling.

**Figure 2:**
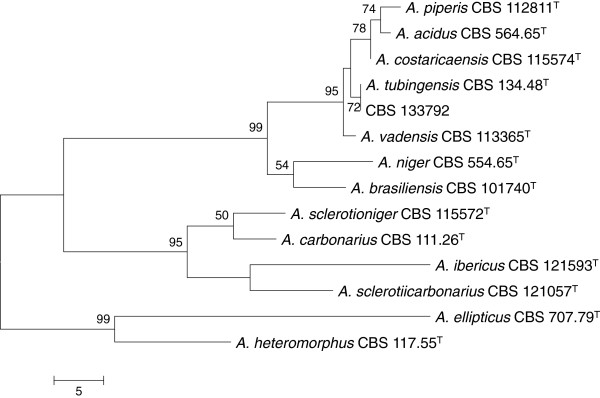
**Maximum Parsimony tree based on β-tubulin sequence data of type strains of *Aspergillus *section *Nigri, *including the *Aspergillus tubingensis *(CBS 133792) strain isolated from the presented patient.** Numbers above branches represent bootstrap values.

Physical examination revealed a body temperature of 37.9°C and normal vital signs. A purulent wound in the mouth resulting from the biopsy, left pre-orbital swelling, and painful cervical lymphadenopathy were observed. Laboratory tests showed increased C-reactive protein at 346 mg/L, leucopenia (leukocytes < 0.1 × 10^9^/L), thrombopenia (thrombocytes 32 × 10^9^/L) and normocytic anaemia (haemoglobin 5.51 × 10^9^/L). Computed tomography of the head showed pre-orbital swelling with induration of the subcutis, swollen mucosa of the sphenoid, and maxillary sinus, and total opacification of the left ethmoid sinus (Figure 1). Since a direct surgical approach was impossible due to extensive bleeding of the swollen mucosa, an antrostomy was performed and sinus secretions were thoroughly aspirated. Cultures of purulent fluid from the biopsy wound in the mouth on Sabouraud dextrose agar at 37.0°C grew a black *Aspergillus*, morphologically resembling *A. niger*. Cultures of the aspirated secretion from sinus cavities were negative for bacteria and fungi. Results of the histological examination of mucosal lining samples of the nasal sinus were consistent with chronic sinusitis. The result of molecular identification of the black *Aspergillus* by sequencing of the β-tubilin gene was *A. tubingensis* Mosseray (CBS-KNAW, Fungal Biodiversity Centre, Utrecht, The Netherlands, strain CBS 133792; GenBank accession number KC163802). Figure 2 shows the phylogenetic relationships among *Aspergillus* section *Nigri* species, including our isolated strain (Table 1). Antifungal susceptibility testing by microdilution showed that the strain had MIC values to voriconazole of 1 μg/mL, to posaconazole of 0.25 μg/mL, to itraconazole of 0.25 μg/mL, to fluconazole of > 24 μg/mL, to anidulafungin of 0.125 μg/mL, to amphothericin B of 0.5 μg/mL, and to flucytosine of 2 μg/mL [7].

**Table 1:** List of Genbank numbers

**Name:**	**GenBank number:**
*A. acidus* CBS 564.65 T	FJ629280
*A. brasiliensis* CBS 101740 T	FJ629272
*A. carbonarius* CBS 111.26 T	FJ629276
*A. costaricaensis* CBS 115574 T	FJ629277
*A. ellipticus* CBS 707.79 T	FJ629279
*A. heteromorphus* CBS 117.55 T	FJ629284
*A. ibericus* CBS 121593 T	AM419748
*A. niger* CBS 554.65 T	FJ629288
*A. piperis* CBS 112811 T	FJ629303
*A. sclerotiicarbonarius* CBS 121057 T	EU159229
*A. sclerotioniger* CBS 115572 T	FJ629304
*A. tubingensis* CBS 134.48 T	FJ629305
CBS 133792	KC163802
*A. vadensis* CBS 113365 T	FJ629319

List of GenBank numbers of isolates from *Aspergillus* section *Nigri* that were used for the Maximum Parsimony tree*,* including the *Aspergillus tubingensis* (CBS 133792) strain isolated from the presented patient.

The patient was treated with liposomal amphothericin B 5 mg/kg for 6 weeks and imipinem 500 mg/qid for 2 weeks intravenously, followed by prolonged treatment with voriconazole 300 mg/tid oral for 4 months. With this treatment the patient recovered from the sinusitis, but passed away later on that year due to BK virus encephalitis.

In summary, we describe an immunocompromised patient suffering from osteomyelitis of the maxillary bone with a probable invasive *A. tubingensis* infection of the surrounding connective tissue after tooth extraction. This probably resulted in dentogenic pansinusitis presenting as an acute ethmoiditis.

## Conclusions

Species belonging to the *Aspergillus* section *Nigri* have been identified as opportunistic pathogens, particularly in cases of otitis and sinusitis. Of the 25 species in section *Nigri*, *A. niger* has most often been described as the cause of infection, however, in most cases species determination was based only on morphology [8]. Osteomyelitis caused by *Aspergillus* section *Nigri* is very rare [9], and by *A. tubingensis* has to the best of our knowledge not been described. We isolated *A. tubingensis* from a purulent wound localized on the surface of the infected maxillary bone. The immunocompromised patient deteriorated while he was treated with antibiotics, and improved after antimycotics were added. Therefore we consider this case as a probable invasive aspergillosis. Alternatively, the infection may have been caused by yeasts, which were noted in the necrotic tissue sampled during the debridement 23 days prior to presentation. We can not rule out involvement of yeasts as well, however, the cultures of the purulent fluid samples taken from the bone biopsy wound at presentation were negative for yeasts.

The subsequent pansinusitis of the patient and acute ethmoiditis were probably dentogenic, since the purulent mouth wound after biopsy was ipsilateral to the pre-orbital swelling. Problematic wound healing of the alveolus after tooth extraction often causes sinusitis due to fistula of the maxillary sinus and contamination with dental microbial flora [10]. Alternatively, *A. tubingensis* could have invaded the sinus and directly cause the sinusitis. However, cultures of aspirated secretion from the sinus cavities were negative, and no fungal strains were found with histological examination of biopsies from the mucosa of the sinus cavities.

In recent studies, clinical isolates presumed as *A. niger* species have been re-classified by sequencing as *A. awamori* and *A. tubingensis* isolates [11-14]. Sequencing of *Aspergillus* isolates from transplant patients with invasive aspergillosis showed that in 9% of cases the causative agent belonged to *Aspergillus* section *Nigri*, and 32% of these isolates were determined as *A. tubingensis*[15]. These studies show the potential of *A. tubingensis* to cause invasive infections.

Differentiating between *A. tubingensis* and *A. niger* also allows for screening for azole resistance. Thus far, studies on susceptibility patterns suggest that these may not be the same for both the species. About 40–50% of *Aspergillus* section *Nigri* strains have a MIC > 1.0 μg/ml to voriconazol, and 70–80% to itraconazol. *A. tubingensis* strains more often have higher MICs to itraconazole and voriconazole [16,17]. Since there are no clinical breakpoints for *Aspergillus* section *Nigri* available*,* it is uncertain how we should interpret these MIC values. Recently, clinical breakpoints have been presented for *A. fumigatus*, proposing MICs ≤ 0.5 μg/ml for voriconazole and itraconazole as susceptible [18]. Therefore, in our hospital we start empirical treatment with liposomal amphothericin B in case of infection with a black *Aspergillus*, and change to azole therapy based on the results of species determination by sequencing and antifungal susceptibility testing.

In conclusion, the presented case shows that *A. tubingensis* may cause severe invasive infections in immunocompromised hosts. Recent developments in species determination within the *Aspergillus* section *Nigri* have resulted in a trend in recognizing *A. tubingensis* as an important opportunistic pathogen. Species determination of clinical *Aspergillus* section *Nigri* isolates by sequencing and antifungal susceptibility testing are both crucial for determining appropriate antifungal therapy, epidemiological data on susceptibility patterns, and the pathogenic potential of the various species.

### Consent

Written informed consent was obtained from the patient for publication of this Case report and any accompanying images. A copy of the written consent is available for review by the Series Editor of this journal.

## Competing interests

The authors declare that they have no competing interests.

## Author’s contributions

EB collected the clinical data and drafted of the manuscript. NS reviewed the diagnostic tools for black *Aspergillus* species and helped with the draft of the manuscript. SS carried out the histological examinations. MM performed the sequencing and provided the Maximum Parsimony tree. HC provided the sequence, contributed to the concept, and edited the manuscript. PH supervised the clinical case interpretation, participated in the coordination and concept of the manuscript, and helped with the draft of the manuscript. All authors read and approved the manuscript.

## Pre-publication history

The pre-publication history for this paper can be accessed here:

http://www.biomedcentral.com/1471-2334/13/59/prepub
